# Associations Between Life-Course Lipid Trajectories and Subclinical Atherosclerosis in Midlife

**DOI:** 10.1001/jamanetworkopen.2022.34862

**Published:** 2022-10-05

**Authors:** Yinkun Yan, Shengxu Li, Yang Liu, Yajun Guo, Camilo Fernandez, Lydia Bazzano, Jiang He, Wei Chen

**Affiliations:** 1 Center for Noncommunicable Disease Management, Beijing Children’s Hospital, Capital Medical University, National Center for Children’s Health, Beijing, China; 2 Department of Epidemiology, Tulane University School of Public Health and Tropical Medicine, New Orleans, Louisiana; 3 Children’s Minnesota Research Institute, Children’s Hospitals and Clinics of Minnesota, Minneapolis; 4 Department of Cardiology, The First Affiliated Hospital of Soochow University, Suzhou, China

## Abstract

**Importance:**

Childhood lipid levels have been associated with adult subclinical atherosclerosis; however, life-course lipid trajectories and their associations with cardiovascular disease risk are poorly characterized.

**Objectives:**

To examine the associations of lipid levels at different ages and discrete lipid trajectory patterns from childhood to adulthood with subclinical atherosclerosis in midlife.

**Design, Setting, and Participants:**

This cohort study used data from the Bogalusa Heart Study, a prospective, population-based cohort study conducted in a semirural, biracial community in Bogalusa, Louisiana, with follow-up from 1973 to 2016 (median follow-up, 36.8 years). Participants had 4 to 16 repeated measurements of lipids, including total cholesterol (TC), non–high-density lipoprotein cholesterol (non-HDL-C), low-density lipoprotein cholesterol (LDL-C), high-density lipoprotein cholesterol (HDL-C), and triglycerides (TG), from childhood to midlife and adult measurement of carotid intima-media thickness (IMT). Statistical analyses were conducted from July 1 to December 31, 2021.

**Exposures:**

Age-specific lipid levels were estimated, and lipid trajectory patterns were identified using latent mixture modeling.

**Main Outcomes and Measures:**

Subclinical atherosclerosis measured by carotid IMT.

**Results:**

The study evaluated 1201 adults (mean [SD] age, 45.7 [6.8] years; 691 [57.5%] women and 510 [42.5%] men; 392 Black [32.6%] and 809 White [67.4%] individuals). Levels of all lipids at each age from 5 to 45 years were significantly associated with adult IMT. The magnitude of associations generally increased with age, and non-HDL-C (age 5 y: β, 0.040; 95% CI, 0.025-0.055; age 45 y, β, 0.049; 95% CI, 0.026-0.072) and LDL-C (age 5 y: β, 0.039; 95% CI, 0.024-0.054; age 45 y, β, 0.043; 95% CI, 0.023-0.063) showed the strongest associations. After adjusting for race, sex, and other cardiovascular risk factors, mean IMT values were significantly higher in the low–slow increase, low–rapid increase, and high-stable trajectory groups for TC (eg, high-stable group: mean difference, 0.152 mm; 95% CI, 0.059-0.244 mm), the low–slow increase, low–rapid increase, moderate-stable, and high–stable trajectory groups for non-HDL-C (eg, low–slow increase group: mean difference, 0.048 mm; 95% CI, 0.012-0.085 mm) and LDL-C (eg, low–rapid increase group: mean difference, 0.104 mm; 95% CI, 0.056-0.151 mm) and the low–rapid increase and moderate-stable trajectory groups for TG (eg, moderate-stable group: mean difference, 0.071 mm; 95% CI, 0.019-0.122 mm) vs the corresponding low-stable trajectory groups. These associations were slightly attenuated after further adjustment for lipid levels at baseline or follow-up. There were no significant differences in mean IMT among HDL-C trajectory groups.

**Conclusions and Relevance:**

In this cohort study, discrete life-course lipid trajectories were associated with the development of atherosclerosis in midlife. The findings emphasize the importance of maintaining optimal lipid levels across the lifespan.

## Key Points

**Question:**

Are lipid trajectory patterns over the life course associated with cardiovascular disease risk in midlife?

**Findings:**

In this cohort study of 1201 participants with multiple lipid measurements from childhood to adulthood, discrete lifetime lipid trajectories were associated with subclinical atherosclerosis in midlife.

**Meaning:**

These results suggest that the longitudinal trajectory patterns of lipid levels could be used to predict future cardiovascular risk; maintaining optimal lipid levels across the lifespan may be warranted to reduce cardiovascular risk.

## Teaser

This cohort study examines the associations of lipid levels at different ages and discrete lipid trajectory patterns from childhood to adulthood with subclinical atherosclerosis in midlife.

## Introduction

Dyslipidemia, characterized by abnormal levels of total cholesterol (TC), non–high-density lipoprotein cholesterol (non-HDL-C), low-density lipoprotein cholesterol (LDL-C), high-density lipoprotein cholesterol (HDL-C), and triglycerides (TG), is a well-established risk factor for atherosclerotic cardiovascular disease.^1,2,3^ Substantial evidence has shown that lipid disorders are associated with subclinical atherosclerosis in youth,^4,5,6^ suggesting that the process of atherosclerosis originates early in life. Childhood lipid disorders have been shown to persist or track into adulthood^7,8^ and thus contribute to the increased risk of cardiovascular disease. Current pediatric guidelines for cardiovascular health and risk reduction by the National Heart, Lung, and Blood Institute recommend universal lipid screening in childhood.^9^ Therefore, identification and intervention of dyslipidemia in early life may prevent and reduce future cardiovascular events.

Previous lifelong studies have demonstrated the association between childhood lipid levels and adult subclinical atherosclerosis.^10,11,12,13,14^ However, most of these studies have relied on single lipid measurements in childhood^13,14^ and thus could not investigate the associations of lipid levels at different ages and changes in lipid levels over time with future atherosclerosis development. Several studies of adults have found that distinct lipid trajectory patterns are associated with risks of cardiovascular disease^15,16^ and kidney disease.^17^ To our knowledge, no prior studies to date have characterized lipid trajectory patterns across the lifespan from young childhood to midlife and evaluated their associations with cardiovascular risk in later life. Using data from the Bogalusa Heart Study with repeated lipid measurements from childhood, we aimed to investigate the associations of lipid levels at different ages as well as discrete lipid trajectory patterns during follow-up with adult subclinical atherosclerosis measured by carotid artery intima-media thickness (IMT).

## Methods

### Study Cohort

The Bogalusa Heart Study is a series of long-term epidemiologic studies in a semirural biracial (35% Black; 65% White) community in Bogalusa, Louisiana, beginning in 1973. This study focuses on the early natural history of cardiovascular disease since childhood.^18^ In the community of Bogalusa, Louisiana, 9 cross-sectional surveys of children aged 4 to 19 years and 12 cross-sectional surveys of adults aged 20 to 58 years who were previously examined as children were conducted between 1973 and 2016. The Bogalusa Heart Study is not a balanced longitudinal study design. It consisted of multiple cross-sectional surveys over 43 years in Bogalusa, with a population ranging from 18 400 in 1973 to 11 300 in 2016. The sample size varied from 1084 to 4222 in the 9 children surveys and from 238 to 1930 in the 12 adult surveys. The total number of different individuals recruited in these surveys was 8385 children and 3780 adults. These adults participated at least once in the children surveys. The exclusion criteria in the current study were nonfasting blood and missing values of lipids in each of the cross-sectional surveys. Linking these repeated cross-sectional examinations conducted every 2 to 3 years has resulted in serial observations from childhood to adulthood in the same individuals. In the longitudinal cohort, 1736 participants had been examined 4 to 16 times for lipids (mean, 7.6 examinations; ≥2 times in childhood and ≥2 times in adulthood). Of these 1736 individuals, 1201 had B-mode ultrasound examination for carotid artery examination during 2004 to 2016. The median follow-up period was 36.8 years, ranging from 19.8 to 39.3 years. The study cohort selection is described in eFigure 1 in the Supplement.

Written informed consent was obtained from each study participant or parent or guardian when appropriate. Study protocols were approved by the institutional review board of the Tulane University Health Sciences Center. This study followed the Strengthening the Reporting of Observational Studies in Epidemiology (STROBE) reporting guideline.

### General Examinations

Standardized protocols were used by trained staff members across all surveys. Height and weight were measured in duplicate, and the mean values were used for analysis. Body mass index (BMI) was calculated as weight in kilograms divided by height in meters squared. Systolic blood pressure (SBP) and diastolic blood pressure (DBP) were recorded using a mercury sphygmomanometer on right arms in a relaxed sitting position by 2 trained observers (3 times each). The mean values of the 6 readings were used for analysis. Adult hypertension was defined as SBP of 140 mm Hg or greater, DBP of 90 mm Hg or greater, or taking antihypertensive medications. Information on smoking and alcohol use was obtained using a staff-administered standardized questionnaire. Current smoking and alcohol drinking were defined as smoking at least 1 cigarette per day and consuming alcohol at least 3 times/wk, respectively, during the prior 12 months.

### Laboratory Measurements

Venous blood samples were collected after fasting for at least 12 hours. During 1973 to 1986, cholesterol and TG levels were measured with a Technicon AutoAnalyzer II (Technicon Instrument Corp). Since 1987, lipid variables were determined by using an Abbott VP instrument (Abbott Laboratories) by enzymatic procedures. Both chemical and enzymatic procedures met the performance requirements of the Lipid Standardization Program of the US Centers for Disease Control and Prevention. Measurements on Centers for Disease Control and Prevention–assigned quality control samples showed no consistent bias over time within or between surveys. Lipoprotein cholesterols were analyzed by using a combination of heparin-calcium precipitation and agar-agarose gel electrophoresis procedures. Non-HDL-C was calculated as TC − HDL-C. Between 1978 and 1991, blood glucose was determined with a glucose oxidase method using a Beckman Glucose Analyzer (Beckman Instruments). Since 1992, glucose was measured enzymatically as part of a multichemistry profile. Diabetes was defined as fasting blood glucose of 126.13 mg/dL (to convert to millimoles per liter, multiply by 0.0555) or taking glucose-lowering medication.^19^

### Carotid Ultrasonography

Carotid artery examination was conducted in adult surveys during 2004 to 2016. If there were 2 or more examinations, the last examination was used in the analysis. Trained sonographers performed ultrasonography examinations with a Sonolayer SSH160A (Toshiba Medical) and a 7.5-MHz linear array transducer on participants in the supine position with the head slightly extended and turned to the opposite direction of the carotid artery being studied. Images were recorded at the common carotid, carotid bulb (bifurcation), and internal carotid arteries bilaterally according to previously developed protocols for the Atherosclerosis Risk in Communities Study.^20^ The mean values of the maximum carotid IMT readings of 3 right and 3 left far walls for common, bulb, and internal segments were used for analysis.

### Statistical Analysis

Log-transformed TG levels (log-TG) were used for analysis. To estimate age-specific lipid levels, random-effects mixed models were used to build lipid growth curves for each individual using multiple lipid measurements during follow-up. This individual-based trajectory model was fitted by SAS Proc Mixed, as previously described.^21,22^ Cubic curves were fitted for all lipid variables in race-sex groups. The lipid levels at each age from 5 to 45 years were estimated based on growth curve parameters for each individual.

We used latent class mixture models to identify distinct subgroups that shared similar underlying trajectories of lipids. This group-based trajectory model was fitted by SAS Proc Traj, as previously described.^23,24,25^ Briefly, multiple lipid data were fitted by the maximum likelihood method as a mixture of multiple latent trajectories in a censored normal model with a polynomial function of age. We tested trajectory models of lipids with the number of trajectory groups ranging from 2 to 5 and the polynomial function of age being the same as that in individual-based models. The optimal model was selected by the maximum bayesian information criterion. We named the trajectories based on baseline lipid levels and the visual change patterns of lipids over time. We then calculated each participant’s posterior predicted probability of belonging to each lipid trajectory group and assigned participants into the trajectory to which their posterior probability of membership was greatest.

Multivariable linear regression analyses were performed to examine the associations of lipid levels at different ages with adult IMT, adjusted for race and sex and age, BMI, lipid-lowering medication, hypertension, diabetes, smoking, and alcohol drinking in the last adult survey. We examined the associations between lipid trajectory patterns and adult IMT using multivariable linear regression analyses adjusted for the covariates mentioned previously. We performed additional analyses with further adjustment for baseline or follow-up lipid levels to investigate how they changed associations. To assess the stability of findings, we did a sensitivity analysis after excluding participants taking lipid-lowering medications.

All analyses were performed using SAS version 9.4 (SAS Institute). A 2-tailed *P* < .05 was considered significant.

## Results

### Participant Characteristics

Of 1201 participants, age ranged between 26 and 57 years (mean [SD] age, 45.7 [6.8] years). Participants comprised 510 (42.5%) men and 691 (57.5%) women, 392 (32.6%) Black and 809 (67.4%) White residents, and 150 (12.5%) participants who reported taking lipid-lowering medications (Table 1).

**Table 1.** Characteristics of 1201 Study Participants

Characteristics	Participants, No. (%) (N = 1201)	
	First childhood survey	Last adulthood survey
Sex		
Female	691 (57.5)	691 (57.5)
Male	510 (42.5)	510 (42.5)
Race		
Black	392 (32.6)	392 (32.6)
White	809 (67.4)	809 (67.4)
Age, mean (SD), y	12.1 (4.5)	45.7 (6.8)
BMI, mean (SD)	19.1 (4.3)	31.0 (7.6)
SBP, mean (SD), mm Hg	103 (11)	122 (16)
DBP, mean (SD), mm Hg	63 (10)	79 (11)
Glucose, mean (SD), mg/dL	87.0 (9.6)	103.9 (39.0)
TC, mean (SD), mg/dL	155.7 (27.0)	194.7 (40.7)
Non-HDL-C, mean (SD), mg/dL	94.1 (27.0)	142.9 (41.8)
LDL-C, mean (SD), mg/dL	86.6 (23.9)	119.5 (37.2)
TG, median (IQR), mg/dL	60.0 (46.0-79.0)	108.0 (76.0-159.0)
HDL-C, mean (SD), mg/dL	61.6 (18.1)	51.8 (16.4)
Lipid-lowering medication	NA	150 (12.5)
Diabetes	NA	186 (15.5)
Hypertension	NA	474 (39.5)
Smoking	NA	351 (29.2)
Alcohol drinking	NA	399 (33.2)
Carotid IMT, mean (SD), mm	NA	0.914 (0.290)

Abbreviations: BMI, body mass index (calculated as weight in kilograms divided by height in meters squared); DBP, diastolic blood pressure; HDL-C, high-density lipoprotein cholesterol; IMT, intima-media thickness; LDL-C, low-density lipoprotein cholesterol; NA, not applicable; non-HDL-C, non–high-density lipoprotein-cholesterol; SBP, systolic blood pressure; TC, total cholesterol; TG, triglycerides.

SI conversion factor: To convert glucose to millimoles per liter, multiply by 0.0555; to convert TC, non-HDL-C, LDL-C, and HDL-C to millimoles per liter, multiply by 0.0259; to convert TG to millimoles per liter, multiply by 0.0113.

### Age-Specific Lipid Levels and Subclinical Atherosclerosis

**Figure 1.**
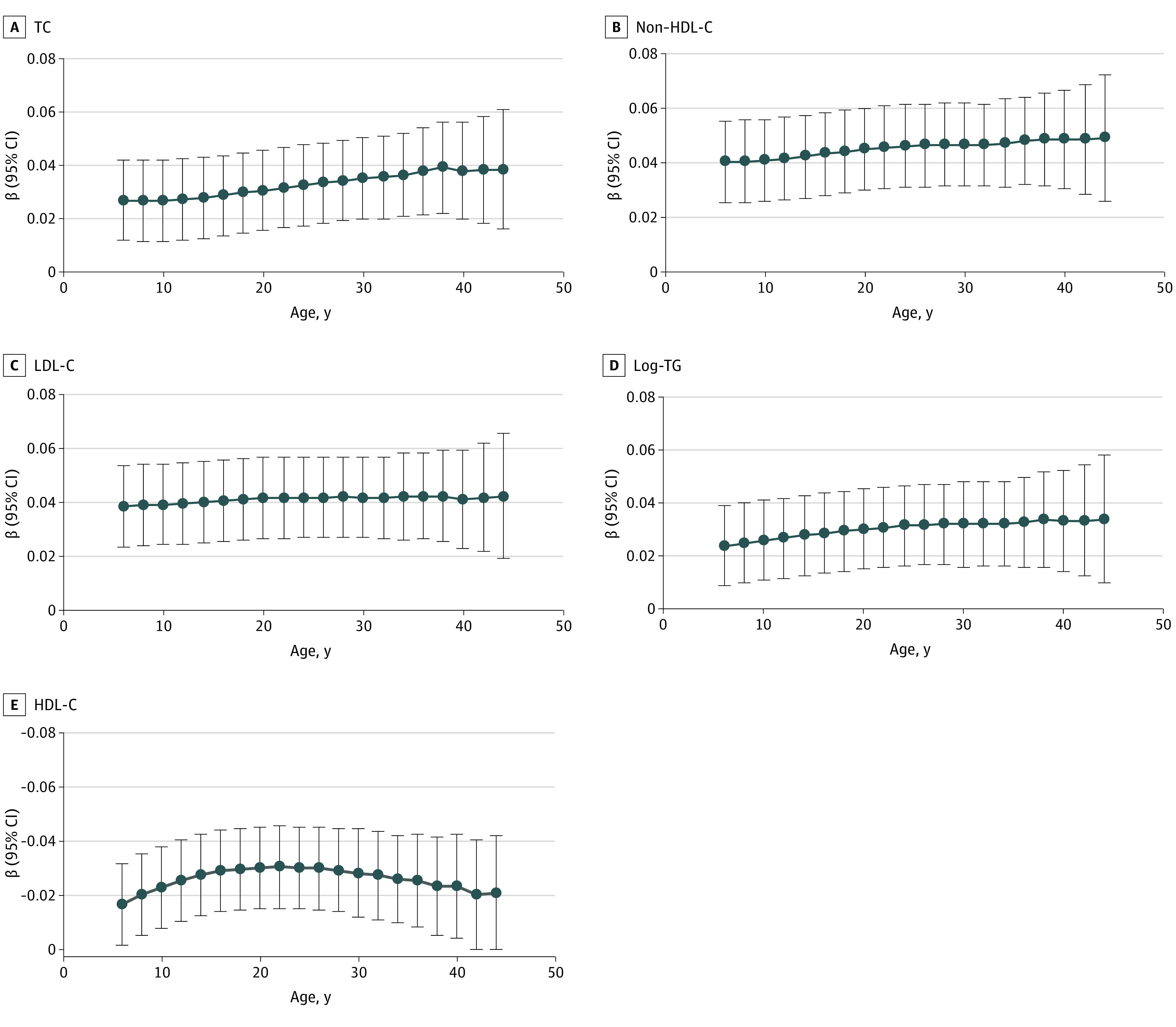
Regression Coefficients for Adult Carotid Intima-Media Thickness per 1-SD Increase in Lipid Levels at Different Ages Analyses were adjusted for race, sex, and age and body mass index, lipid-lowering medication, hypertension, diabetes, smoking, and alcohol drinking in the last adult survey. HDL-C indicates high-density lipoprotein cholesterol; LDL-C, low-density lipoprotein cholesterol; non-HDL-C, non–high-density lipoprotein-cholesterol; TC, total cholesterol; and TG, triglycerides.

Growth curves of lipids from childhood to adulthood by race and sex are shown in eFigure 2 in the Supplement. As shown in Figure 1, after adjustment for race, sex, and age, BMI, lipid-lowering medication, hypertension, diabetes, smoking, and alcohol drinking in the last adult survey, levels of TC, non-HDL-C, LDL-C, and log-TG at all ages were significantly and positively associated with adult IMT, and the β values for an 1-SD increase of lipid levels steadily increased from age 5 years to age 45 years, ranging from 0.027 (95% CI, 0.012-0.042) to 0.039 (95% CI, 0.016-0.061) for TC, from 0.040 (95% CI, 0.025-0.055) to 0.049 (95% CI, 0.026-0.072) for non-HDL-C, from 0.039 (95% CI, 0.024-0.054) to 0.043 (95% CI, 0.023-0.063) for LDL-C, and from 0.023 (95% CI, 0.008-0.039) to 0.033 (95% CI, 0.010-0.057) for log-TG. In contrast, the levels of HDL-C at all age points were significantly and inversely associated with adult IMT, and the absolute β values steadily increased from 0.017 (95% CI, 0.002-0.032) at age 5 years to 0.031 (95% CI, 0.015-0.046) at age 20 years, and then showed a slight decrease.

### Lipid Trajectory Groups

**Figure 2.**
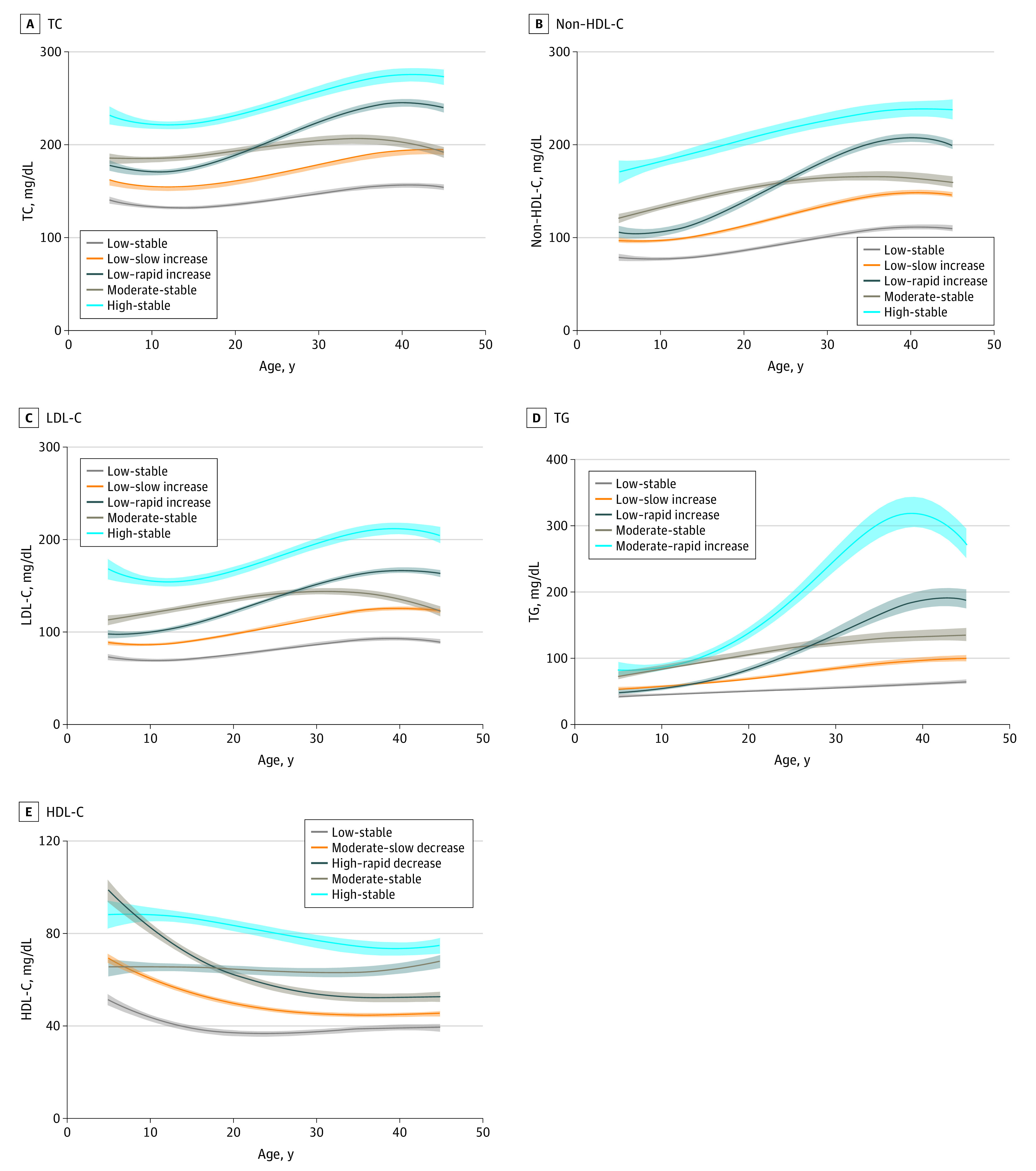
Lipid Trajectory Groups From Childhood to Adulthood To convert total cholesterol (TC), non–high-density lipoprotein-cholesterol (non-HDL-C), low-density lipoprotein cholesterol (LDL-C), and high-density lipoprotein cholesterol (HDL-C) to millimoles per liter, multiply by 0.0259; triglycerides (TG) to millimoles per liter, multiply by 0.0113.

Using group-based trajectory modeling, we identified 5 discrete trajectories from age 5 to 45 years separately for all lipid variables (Figure 2). The trajectory groups were named according to their lipid levels at baseline and trends over time (ie, stable, decrease, and increase). For TC, non-HDL-C, and LDL-C, the 5 five trajectory patterns were labeled (1) low-stable, characterized by maintaining low lipid levels throughout follow-up; (2) low–slow increase, starting with a low lipid level and experiencing a slow increase beginning at approximately age 12 years; (3) low–rapid increase, starting with a low lipid level and experiencing a rapid increase beginning at approximately age 12 years; (4) moderate-stable, maintaining moderate lipid levels throughout follow-up; and (5) high-stable, maintaining high lipid levels throughout follow-up. For TG, the 5 discrete trajectory patterns were labeled low-stable, low–slow increase, low–rapid increase, moderate-stable, and moderate–rapid increase (characterized by starting with a moderate lipid level and then experiencing a rapid increase). For HDL-C, the 5 discrete trajectories were labeled low-stable, moderate–slow decrease (characterized by starting with a moderate lipid level and then experiencing a slow decrease), moderate–slow increase (characterized by starting with a moderate lipid level and then experiencing a slow increase), high–rapid decrease (starting with a high lipid level and experiencing a rapid decrease), and high-stable. Among these trajectory patterns, the low–slow increase groups of TC, non-HDL-C, LDL-C, and TG and the moderate–slow decrease group of HDL-C accounted for the largest proportions; the high-stable groups of TC, non-HDL-C, LDL-C, and HDL-C and the moderate–rapid increase group of TG accounted for the smallest proportions (Table 2). Men were more likely to be categorized into low–rapid increase groups of TC, non-HDL-C, LDL-C, and TG and low-stable group of HDL-C. White participants were more likely to be categorized into low–rapid increase group of TC, high-stable groups of non-HDL-C and LDL-C, moderate–rapid increase group of TG, and low-stable group of HDL-C. Most clinical variables at baseline and follow-up were significantly different among lipid trajectory groups (eTables 1-5 in the Supplement).

**Table 2.** Covariate-Adjusted Means of Adult Carotid IMT by Lipid Trajectory Group

Trajectory group	Participants, No. (%)	Model 1^a^		Model 2^b^	
		IMT, Mean (SE), mm	*P* value	IMT, mean (SE), mm	*P* value
TC					
Low-stable	265 (22.1)	0.887 (0.017)	NA	0.893 (0.020)	NA
Low–slow increase	556 (46.3)	0.932 (0.012)	.02	0.932 (0.013)	.06
Low–rapid increase	173 (14.4)	0.952 (0.021)	.01	0.949 (0.021)	.06
Moderate-stable	172 (14.3)	0.926 (0.021)	.13	0.920 (0.023)	.40
High-stable	35 (2.9)	1.039 (0.045)	<.001	1.025 (0.050)	.02
Non-HDL-C					
Low-stable	316 (26.3)	0.876 (0.016)	NA	0.877 (0.018)	NA
Low–slow increase	561 (46.7)	0.924 (0.012)	.009	0.924 (0.012)	.02
Low–rapid increase	143 (11.9)	0.972 (0.023)	<.001	0.972 (0.023)	.002
Moderate-stable	165 (13.7)	0.988 (0.021)	<.001	0.985 (0.025)	.002
High-stable	16 (1.3)	1.107 (0.066)	<.001	1.101 (0.072)	.004
LDL-C					
Low-stable	299 (24.9)	0.867 (0.016)	NA	0.875 (0.018)	NA
Low–slow increase	550 (45.8)	0.935 (0.012)	<.001	0.936 (0.012)	.003
Low–rapid increase	199 (16.6)	0.970 (0.019)	<.001	0.965 (0.020)	.001
Moderate-stable	134 (11.2)	0.959 (0.023)	<.001	0.944 (0.027)	.05
High-stable	19 (1.6)	1.034 (0.060)	.007	1.009 (0.065)	.06
TG					
Low-stable	237 (19.7)	0.885 (0.018)	NA	0.894 (0.020)	NA
Low–slow increase	505 (42.1)	0.927 (0.013)	.05	0.929 (0.013)	.11
Low–rapid increase	191 (15.9)	0.946 (0.020)	.03	0.947 (0.020)	.06
Moderate-stable	202 (16.8)	0.956 (0.020)	.007	0.945 (0.021)	.09
Moderate–rapid increase	66 (5.5)	0.942 (0.034)	.15	0.928 (0.036)	.43
HDL-C					
Low-stable	226 (18.8)	0.962 (0.019)	.08	0.958 (0.022)	.16
Moderate–slow decrease	606 (50.5)	0.933 (0.012)	.23	0.933 (0.012)	.31
Moderate-stable	137 (11.4)	0.918 (0.023)	.46	0.919 (0.024)	.52
High–rapid decrease	182 (15.2)	0.889 (0.020)	.93	0.892 (0.022)	.96
High-stable	50 (4.2)	0.886 (0.038)	NA	0.890 (0.040)	NA

Abbreviations: HDL-C, high-density lipoprotein cholesterol; IMT, intima-media thickness; LDL-C, low-density lipoprotein cholesterol; NA, not applicable; non-HDL-C, non–high-density lipoprotein-cholesterol; SE, standard errors; TC, total cholesterol; TG, triglycerides;

^a^ Model 1 was adjusted for race and sex and age, body mass index, lipid-lowering medication, hypertension, diabetes, smoking, and alcohol drinking in the last adult survey.

^b^ Model 2 was additionally adjusted for baseline lipid levels.

### Lipid Trajectory Groups and Subclinical Atherosclerosis

**Figure 3.**
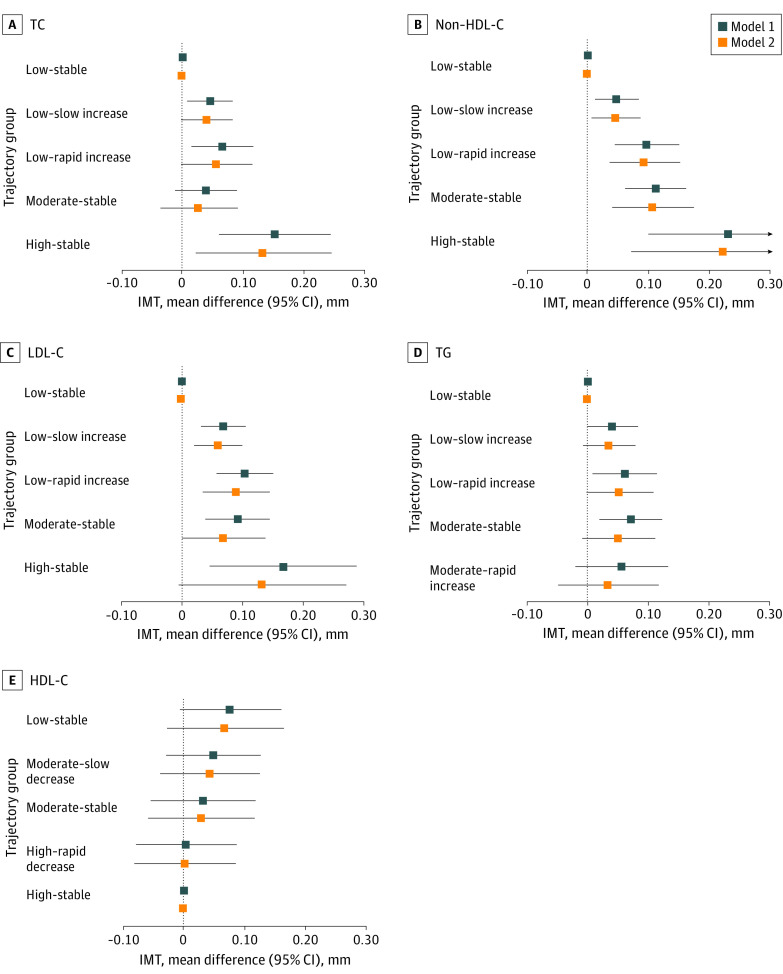
Mean Differences of Adult Carotid Intima-Media Thickness (IMT) Among Lipid Trajectory Groups Model 1 was adjusted for age, race, sex, and body mass index, lipid-lowering medication, hypertension, diabetes, smoking, and alcohol drinking in the last adult survey. Model 2 was additionally adjusted for baseline lipid levels. HDL-C indicates high-density lipoprotein cholesterol; LDL-C, low-density lipoprotein cholesterol; non-HDL-C, non-high-density lipoprotein-cholesterol; TC, total cholesterol; and TG, triglycerides.

As shown in Table 2 and Figure 3, after adjusting for race, sex, age, and BMI, lipid-lowering medication, hypertension, diabetes, smoking, and alcohol drinking in the last adult survey, compared with the low-stable group, the adjusted mean values of adult IMT were significantly higher in low–slow increase, low–rapid increase, and high-stable groups of TC (eg, high-stable group: mean difference, 0.152 mm; 95% CI, 0.059-0.244 mm), all 4 adverse trajectory groups of non-HDL-C (eg, low–slow increase group: mean difference, 0.048 mm; 95% CI, 0.012-0.085 mm) and LDL-C (eg, low–rapid increase group: mean difference, 0.104 mm; 95% CI, 0.056-0.151 mm), and the low–rapid increase and moderate-stable groups of TG (eg, moderate-stable group: mean difference, 0.071 mm; 95% CI, 0.019-0.122 mm). Moreover, the high-stable group of TC had significantly higher levels of carotid IMT than the low–slow increase group (mean difference, 0.107 mm; 95% CI, 0.018-0.196 mm) and the moderate-stable group (mean difference, 0.113 mm; 95% CI, 0.018-0.207 mm); the high-stable group (mean difference, 0.183 mm; 95% CI, 0.052-0.313 mm) and moderate-stable group (mean difference, 0.063 mm; 95% CI, 0.018-0.109 mm) of non-HDL-C had significantly higher levels of carotid IMT vs the low–slow increase group. There were no significant differences in the adjusted mean values of adult IMT among the HDL-C trajectory groups (eTable 6 in the Supplement).

After further adjustment for baseline lipid levels, the associations between lipid trajectory groups and IMT were slightly attenuated. The adjusted mean values of adult IMT remained significantly higher in the high-stable group of TC, all 4 adverse groups of non-HDL-C, and low–slow increase and low–rapid increase groups of LDL-C. There were no significant differences in the adjusted mean values of adult IMT among TG and HDL-C trajectory groups (Table 2 and Figure 3). Similar results were found when adjusting for adult lipid levels at follow-up instead of childhood levels at baseline (eTable 7 in the Supplement). There were no significant changes in *R*^2^ values after incorporating childhood lipid levels (TC: changes in *R*^2^ = 0.0018; *P* = .14; non-HDL-C: changes in *R*^2^ = 0.0001; *P* = .83; LDL-C: changes in *R*^2^ = 0.0002; *P* = .61; TG: *R*^2^ = 0.0002; *P* = .59; HDL-C: *R*^2^ = 0.0003; *P* = .50). In contrast, the changes in *R*^2^ values were significant after including adult lipid levels into the trajectory-IMT association models for TC (changes in *R*^2^ = 0.013; *P* < .001), non-HDL-C (changes in *R*^2^ = 0.006; *P* = .007), and LDL-C (changes in *R*^2^ = 0.004; *P* = .02) but not for TG (changes in *R*^2^ = 0.0002; *P* = .58) and HDL-C (changes in *R*^2^ = 0.0004; *P* = .45).

Most associations between lipid trajectory and adult IMT slightly changed after excluding 150 participants taking lipid-lowering medications. Details appear in eTable 8 in the Supplement.

## Discussion

This longitudinal cohort study with follow up from childhood into middle adulthood found that lipid levels at each age during follow-up were significantly associated with adult subclinical atherosclerosis, and the strength of these associations generally increased with age. The 5 distinct life-course lipid trajectories we identified, particularly trajectories of non-HDL-C and LDL-C, were associated with subclinical atherosclerosis development later in midlife beyond other cardiovascular risk factors. Notably, most of these associations were independent of lipid levels at baseline or follow-up. These findings underscore the importance of maintaining optimal lipid levels from early life for long-term cardiovascular health benefits.

It is well documented that the atherosclerotic process begins in childhood.^4,26^ Previous studies, including our own, have found that individual lipid levels in childhood or time-averaged lipid levels during follow-up are significantly and independently associated with adult IMT.^11,12,27,28^ In the current study, we extended the previous studies by estimating age-specific lipid levels during ages 5 to 45 years for each individual based on serial lipid measurements and found that the levels of all lipids at each age were significantly associated with adult IMT. Moreover, the strength of these associations generally increased with age. These observations are consistent with previous findings that childhood TC and non-HDL-C levels were significantly associated with adult subclinical atherosclerosis.^28,29^ Additionally, we found that LDL-C and non-HDL-C levels had stronger associations with adult IMT than other lipid measures. In line with these findings, many studies in adults have shown LDL-C and non-HDL-C levels are more strongly associated with risks of cardiovascular mortality^30,31^ than other lipid parameters. Indeed, the adult guidelines for dyslipidemia management recommend LDL-C as the primary treatment target and non-HDL-C as the secondary therapy target.^32^ Our findings provide strong evidence that universal screening for lipid disorders should be conducted from early life, particularly for non-HDL-C and LDL-C.

Despite strong child-to-adult tracking of lipids,^7,8^ heterogeneous life-course trajectories may exist. The Cardiovascular Risk in Young Finns Study has identified several trajectories of lipids across childhood and early adulthood that are associated with the risk of later-life depressive symptoms in 824 participants,^33^ but the lipid trajectories may be less accurate because only 4 measurements of lipids were available. In contrast, we have collected up to 16 lipid measurements during 36.8 years of follow-up and identified 5 distinct trajectory groups for lipids using latent class mixture modeling. These group-based trajectories account for baseline values, variations, and long-term trends over time; distinguish heterogeneity within individuals; and provide a more realistic understanding of longitudinal changes in lipid levels during lifetime.^23^ Nevertheless, the determinants of these trajectories are not fully understood, and future studies are required to examine the influences of genetic, lifestyle, and biological factors on the heterogeneous development of lipid disorders.

Previous studies have shown that lipid trajectories during adulthood are associated with risks of cardiovascular and kidney outcomes.^15,16,17^ Our previous publications have demonstrated that changes in dyslipidemia status of LDL-C and HDL-C between adolescence and adulthood were associated with adult IMT.^29^ A recent study found that individuals who resolve their non-HDL-C dyslipidemia by adulthood have normalized risk of developing high carotid IMT in adulthood.^28^ In these studies, lipid changes were calculated as differences between first childhood and last adult measurements and thus did not reflect the age-related lipid trajectory patterns.

In the current study, we found that life-course trajectories of lipids (except for HDL-C) were associated with adult preclinical atherosclerosis independent of race, sex, and other cardiovascular risk factors. Compared with lipid trajectory groups with consistently low lipid levels, the mean values of adult IMT were significantly higher in most trajectory groups that had moderate or high levels of TC, non-HDL, LDL-C and TG. Importantly, participants who had low lipid levels in early life but showed an increase during follow-up also had higher adult IMT, suggesting a critical deleterious effect of adverse lipid changes on adult atherosclerosis development. Most of these associations between lipid trajectory and adult IMT slightly changed after further adjustment for lipid levels at baseline or follow-up, suggesting that life-course lipid trajectories characterized by serially repeated measurements provide additional information beyond single lipid measurements. Previous extensive evidence has shown that life-course trajectory patterns of BMI and blood pressure are also significantly associated with adult cardiovascular risk.^34,35,36,37^ All these findings together suggest that longitudinal measurements of lipids and other cardiovascular risk factors starting from early life may be important for identifying individuals at high risk for further cardiovascular disease.

### Strengths and Limitations

This study has strengths. The community-based longitudinal study cohort with repeated lipid measurements from childhood to adulthood provides a unique opportunity to examine the association of lipid levels at specific age points as well as lipid trajectory patterns during follow-up with adult subclinical atherosclerosis.

This study also has several limitations. First, our study is a biracial cohort of Black and White participants. The identified trajectory groups may not be generalizable to other populations. Second, individuals under pharmacological treatments targeting dyslipidemia represent a subgroup who, without treatment, would be expected to have the most adverse lipid levels. This may result in bias in trajectory and association analyses to some extent. For that reason, we performed a sensitivity analysis by excluding participants with pharmacological treatments, and the results remained nearly unchanged. Third, the sample size was not sufficiently large for analyses by subgroups, especially Black men, and thus race- and sex-specific trajectory analyses were not performed. Large-scale longitudinal studies with multiple ethnic groups are needed to confirm the findings of the current study.

## Conclusions

This study found that lipid levels beginning in childhood as well as distinct lipid trajectories over the life course are associated with subclinical atherosclerosis in midlife. The findings suggest that screening for dynamic changes in lipid profiles from early life may improve identification of high-risk individuals and prevention of cardiovascular disease.

## Notes

Supplement. **eTable 1. **Characteristics by TC Trajectory Group **eTable 2. **Characteristics by Non-HDL-C Trajectory Group **eTable 3. **Characteristics by LDL-C Trajectory Group **eTable 4. **Characteristics by TG Trajectory Group **eTable 5. **Characteristics by HDL-C Trajectory Group **eTable 6. **Covariate-Adjusted Carotid IMT and *P *Values for Comparisons Between Lipid Trajectory Groups **eTable 7. **Covariate-Adjusted Means of Adult Carotid IMT by Lipid Trajectory Group Adjusted for Clinical Risk Factors and Lipid Levels at Follow-up **eTable 8. **Covariate-Adjusted Means of Adult Carotid IMT by Lipid Trajectory Group After Excluding Participants With Lipid-Lowing Medications **eFigure 1. **Flow Chart of the Study Cohort Selection **eFigure 2. **Growth Curves of Lipids by Race and Sex
