# Supplementary material for: Associations Between Life-Course Lipid Trajectories and Subclinical Atherosclerosis in Midlife
Source: JAMA Netw Open. 2022 Oct 5;5(10):e2234862. doi: 10.1001/jamanetworkopen.2022.34862 (PMC9535509; doi:10.1001/jamanetworkopen.2022.34862)
Supplement: Supplement. — eTable 1. Characteristics by TC Trajectory Group eTable 2. Characteristics by Non-HDL-C Trajectory Group eTable 3. Characteristics by LDL-C Trajectory Group eTable 4. Characteristics by TG Trajectory Group eTable 5. Characteristics by HDL-C Trajectory Group eTable 6. Covariate-Adjusted Carotid IMT and P Values for Comparisons Between Lipid Trajectory Groups eTable 7. Covariate-Adjusted Means of Adult Carotid IMT by Lipid Trajectory Group Adjusted for Clinical Risk Factors and Lipid Levels at Follow-up eTable 8. Covariate-Adjusted Means of Adult Carotid IMT by Lipid Trajectory Group After Excluding Participants With Lipid-Lowing Medications eFigure 1. Flow Chart of the Study Cohort Selection eFigure 2. Growth Curves of Lipids by Race and Sex [file jamanetwopen-e2234862-s001.pdf]

Supplemental Online Content

Yan Y, Li S, Liu Y, et al. Associations between life-course lipid trajectories and subclinical atherosclerosis in midlife. *JAMA Netw Open.* 2022;5(10):e2234862. doi:10.1001/jamanetworkopen.2022.34862

eTable 1. Characteristics by TC Trajectory Group

eTable 2. Characteristics by Non-HDL-C Trajectory Group

eTable 3. Characteristics by LDL-C Trajectory Group

eTable 4. Characteristics by TG Trajectory Group

eTable 5. Characteristics by HDL-C Trajectory Group

eTable 6. Covariate-Adjusted Carotid IMT and *P* Values for Comparisons Between Lipid Trajectory Groups

eTable 7. Covariate-Adjusted Means of Adult Carotid IMT by Lipid Trajectory Group Adjusted for Clinical Risk Factors and Lipid Levels at Follow-up

eTable 8. Covariate-Adjusted Means of Adult Carotid IMT by Lipid Trajectory Group After Excluding Participants With Lipid-Lowing Medications

eFigure 1. Flow Chart of the Study Cohort Selection

eFigure 2. Growth Curves of Lipids by Race and Sex

This supplemental material has been provided by the authors to give readers additional information about their work.

© 2022 Yan Y et al. *JAMA Network Open.*

eTable 1. Characteristics by TC Trajectory Group

Characteristics	Low-Stable (n=265)	Low-Slow Increase (n=556)	Low-Rapid Increase (n=173)	Moderate-Stable (n=172)	High-Stable (n=35)	P
White, n (%)	173 (65.3)	384 (69.1)	132 (76.3)	100 (58.1)	20 (57.1)	0.003
Male, n (%)	119 (44.9)	226 (40.6)	89 (51.4)	62 (36.0)	14 (40.0)	0.04
Childhood						
Age, mean (SD), y	12.4 (4.8)	12.2 (4.4)	12.7 (4.2)	11.0 (4.4)	11.0 (3.4)	0.003
BMI, mean (SD), kg/m^2	19.1 (4.3)	19.0 (4.2)	19.6 (4.6)	18.9 (4.2)	18.9 (3.2)	0.51
SBP, mean (SD), mm Hg	103 (11)	102.4 (11)	104 (10)	101 (11)	105 (13)	0.12
DBP, mean (SD), mm Hg	64 (10)	63.1 (10)	64 (9)	62 (10)	64 (10)	0.27
Glucose, mean (SD), mg/dL	86.3 (8.2)	86.7 (8.5)	87.9 (8.5)	87.4 (14.9)	89.1 (7.0)	0.25
TC, mean (SD), mg/dL	130.9 (17.6)	152.0 (17.5)	168.0 (20.3)	182.1 (23.2)	212.4 (31.1)	<0.001
non-HDL-C, mean (SD), mg/dL	73.4 (17.9)	90.9 (20.1)	106.6 (24.9)	114.5 (27.8)	137.5 (34.9)	<0.001
LDL-C, mean (SD), mg/dL	67.5 (15.9)	83.6 (17.4)	97.8 (20.8)	106.1 (23.8)	127.9 (31.6)	<0.001
TG, median (IQR), mg/dL	53.0 (42.0-68.0)	59.0 (47.0-77.0)	65.0 (50.0-88.0)	66.5 (50.0-84.0)	64.0 (52.0-91.0)	<0.001
HDL-C, mean (SD), mg/dL	57.4 (16.5)	61.1 (16)	61.4 (20)	67.6 (20.8)	74.9 (24.2)	<0.001
Adulthood						
Age, mean (SD), y	45.5 (7.2)	46.1 (6.7)	46.1 (6.2)	44.5 (6.7)	45.5 (6.6)	0.08
BMI, mean (SD), kg/m^2	30.5 (8)	30.7 (7.3)	31.3 (7.1)	31.8 (8)	33.3 (8.0)	0.11
SBP, mean (SD), mm Hg	122 (17)	122 (17)	121 (13)	122 (17)	123 (17)	0.91
DBP, mean (SD), mm Hg	78 (11)	79 (11)	79 (9)	79 (12)	81 (10)	0.75
Glucose, mean (SD), mg/dL	103 (36.6)	101.4 (32.3)	110.4 (56.1)	105.5 (38.1)	110.3 (51.6)	0.08
TC, mean (SD), mg/dL	159.4 (26.7)	195.1 (31.4)	233.7 (39.2)	194.9 (32.6)	257.4 (54.2)	<0.001
non-HDL-C, mean (SD), mg/dL	110.6 (27.8)	142.6 (34.6)	181.5 (40.7)	142.9 (34.9)	199.0 (56.5)	<0.001
LDL-C, mean (SD), mg/dL	88.9 (24.2)	119.2 (29.1)	152.6 (32.8)	123.4 (30.1)	176.2 (65.8)	<0.001
TG, median (IQR), mg/dL	90.0 (67-131)	109.0 (76.0-153.0)	151.0 (95.0-226.0)	102.5 (76.0-143.0)	153.0 (99.0-252.0)	<0.001
HDL-C, mean (SD), mg/dL	48.5 (14.7)	51.3 (15.7)	51 (16.3)	51.9 (15.6)	57.3 (14.3)	0.009
Lipid-lowing Medication, n (%)	13 (4.9)	49 (8.8)	37 (21.4)	40 (23.3)	11 (31.4)	<0.001
Hypertension, n (%)	101 (38.1)	206 (37.1)	70 (40.5)	78 (45.3)	19 (54.3)	0.12
Diabetes, n (%)	40 (15.1)	77 (13.8)	33 (19.1)	31 (18.0)	5 (14.3)	0.43
Smoking, n (%)	74 (27.9)	163 (29.3)	59 (34.1)	47 (27.3)	8 (22.9)	0.53
Alcohol drinking, n (%)	90 (34.0)	194 (34.9)	60 (34.7)	49 (28.5)	6 (17.1)	0.15
Carotid IMT, mean (SD), mm	0.871 (0.258)	0.918 (0.294)	0.958 (0.290)	0.897 (0.289)	1.033 (0.380)	0.002

Abbreviations: BMI, body mass index; SBP, systolic blood pressure; DBP, diastolic blood pressure; TC, total cholesterol; non-HDL-C, Non-high-density lipoprotein-cholesterol; LDL-C, low-density lipoprotein cholesterol; HDL-C, high-density lipoprotein cholesterol; TG, triglycerides; IMT, intima-media thickness.

SI conversion factor: To convert glucose to mmol/L, multiply by 0.0555; to convert TC, non-HDL-C, LDL-C, and HDL-

© 2022 Yan Y et al. *JAMA Network Open.*

C to mmol/L, multiply by 0.0259; to convert TG to mmol/L, multiply by 0.0113.

One-way ANOVA and chi-square tests were performed for continuous variables and categorical variables, respectively.

© 2022 Yan Y et al. *JAMA Network Open.*

eTable 2. Characteristics by Non-HDL-C Trajectory Group

Characteristics	Low-Stable (n=316)	Low-Slow Increase (n=561)	Low-Rapid Increase (n=143)	Moderate-Stable (n=165)	High-Stable (n=16)	P
White, n (%)	190 (60.1)	378 (67.4)	109 (76.2)	119 (72.1)	13 (81.3)	0.003
Male, n (%)	127 (40.2)	227 (40.5)	91 (63.6)	60 (36.4)	5 (31.3)	<0.001
Childhood						
Age, mean (SD), y	12.6 (4.7)	12.0 (4.4)	12.8 (3.8)	10.9 (4.4)	12.3 (6.6)	<0.001
BMI, mean (SD), kg/m^2	18.8 (4.2)	19 (4.2)	19.3 (4.1)	19.6 (4.8)	21.9 (5.4)	0.02
SBP, mean (SD), mm Hg	103 (11)	103 (11)	104 (11)	102 (11)	107 (9)	0.15
DBP, mean (SD), mm Hg	64 (10)	63 (10)	65 (9)	62 (10)	64 (11)	0.04
Glucose, mean (SD), mg/dL	86.2 (8.5)	87.0 (8.8)	88.1 (8.3)	87.1 (14.3)	91.6 (6.6)	0.11
TC, mean (SD), mg/dL	136.8 (20.1)	154.2 (20.6)	163.7 (22.0)	183.8 (24.5)	222.8 (35.8)	<0.001
non-HDL-C, mean (SD), mg/dL	72.5 (16.4)	91.9 (17.7)	102.6 (19.0)	128.3 (25.3)	168 (31.1)	<0.001
LDL-C, mean (SD), mg/dL	67.3 (14.9)	84.9 (16.0)	94.4 (17.1)	116.6 (21.8)	150.3 (30.0)	<0.001
TG, median (IQR), mg/dL	52.0 (41.0-65.0)	59.0 (46.0-76.0)	65.0 (51.0-86.0)	78.0 (57.0-106.0)	91.5 (65.0-126.0)	<0.001
HDL-C, mean (SD), mg/dL	64.3 (16.7)	62.3 (17.2)	61.1 (18.4)	55.5 (21.1)	54.7 (24.9)	<0.001
Adulthood						
Age, mean (SD), y	46.2 (6.9)	45.9 (6.8)	46.1 (6.5)	43.9 (6.8)	45.5 (4.7)	0.008
BMI, mean (SD), kg/m^2	29.4 (7.0)	31.2 (7.7)	31.7 (7.3)	32.0 (8.0)	36.2 (7.0)	<0.001
SBP, mean (SD), mm Hg	122 (17)	122 (17)	123 (14)	121 (16)	129 (17)	0.31
DBP, mean (SD), mm Hg	78 (11)	79 (11)	80 (9)	79 (12)	84 (10)	0.09
Glucose, mean (SD), mg/dL	98.7 (28.5)	103.1 (35.8)	112.4 (57.0)	105.6 (41.2)	138.6 (69.5)	<0.001
TC, mean (SD), mg/dL	166.1 (28.4)	195.2 (32.9)	229.2 (43.9)	210 (36.6)	281.7 (66.6)	<0.001
non-HDL-C, mean (SD), mg/dL	109.2 (26.6)	143.4 (31.9)	183.4 (44.3)	162.2 (34.4)	237.4 (62.4)	<0.001
LDL-C, mean (SD), mg/dL	89.3 (22.6)	121.4 (29.1)	151.9 (35.6)	135.9 (30.5)	212.3 (83.4)	<0.001
TG, median (IQR), mg/dL	84.5 (63.0-113.5)	111.0 (77.0-155.0)	170.0 (107.0-241.0)	125.5 (89.5-185.5)	187.0 (114.5-293.5)	<0.001
HDL-C, mean (SD), mg/dL	55.8 (17.5)	50.7 (14.9)	45.6 (12.2)	47.5 (14.2)	43.7 (12.1)	<0.001
Lipid-lowing Medication, n (%)	15 (4.7)	57 (10.2)	39 (27.3)	33 (20.0)	6 (37.5)	<0.001
Hypertension, n (%)	114 (36.1)	217 (38.7)	61 (42.7)	69 (41.8)	13 (81.3)	0.006
Diabetes, n (%)	37 (11.7)	83 (14.8)	27 (18.9)	34 (20.6)	5 (31.3)	0.02
Smoking, n (%)	82 (25.9)	171 (30.5)	47 (32.9)	43 (26.1)	8 (50.0)	0.12
Alcohol drinking, n (%)	114 (36.1)	187 (33.3)	48 (33.6)	47 (28.5)	3 (18.8)	0.37
Carotid IMT, mean (SD), mm	0.856 (0.211)	0.910 (0.287)	0.987 (0.328)	0.947 (0.338)	1.158 (0.475)	<0.001

Abbreviations: BMI, body mass index; SBP, systolic blood pressure; DBP, diastolic blood pressure; TC, total cholesterol; non-HDL-C, Non-high-density lipoprotein-cholesterol; LDL-C, low-density lipoprotein cholesterol; HDL-C, high-density lipoprotein cholesterol; TG, triglycerides; IMT, intima-media thickness.

SI conversion factor: To convert glucose to mmol/L, multiply by 0.0555; to convert TC, non-HDL-C, LDL-C, and HDL-

© 2022 Yan Y et al. *JAMA Network Open.*

C to mmol/L, multiply by 0.0259; to convert TG to mmol/L, multiply by 0.0113.

One-way ANOVA and chi-square tests were performed for continuous variables and categorical variables, respectively.

© 2022 Yan Y et al. *JAMA Network Open.*

eTable 3. Characteristics by LDL-C Trajectory Group

Characteristics	Low-Stable (n=299)	Low-Slow Increase (n=550)	Low-Rapid Increase (n=199)	Moderate-Stable (n=134)	High-Stable (n=19)	P
White, n (%)	182 (60.9)	376 (68.4)	145 (72.9)	92 (68.7)	14 (73.7)	0.06
Male, n (%)	123 (41.1)	224 (40.7)	104 (52.3)	51 (38.1)	8 (42.1)	0.04
Childhood						
Age, mean (SD), y	12.4 (4.6)	12.2 (4.6)	12.5 (4.0)	10.9 (4.3)	11.3 (3.7)	0.009
BMI, mean (SD), kg/m^2	18.8 (4.0)	19 (4.3)	19.6 (4.4)	19.2 (4.4)	19.7 (3.8)	0.28
SBP, mean (SD), mm Hg	103 (12)	103 (11)	104 (11)	102 (11)	103 (10)	0.35
DBP, mean (SD), mm Hg	64 (10)	63 (10)	64 (10)	62 (10)	63 (10)	0.31
Glucose, mean (SD), mg/dL	86.3 (8.5)	87.2 (10.6)	87.5 (8.8)	86.4 (8.8)	90.6 (6.2)	0.23
TC, mean (SD), mg/dL	136.1 (20.2)	152.7 (20.0)	167.9 (21.7)	185.4 (24.7)	215.1 (37.3)	<0.001
non-HDL-C, mean (SD), mg/dL	73.2 (18.4)	90.8 (18.0)	105.4 (21.0)	128.9 (26.1)	153.1 (36.8)	<0.001
LDL-C, mean (SD), mg/dL	67.2 (16.0)	83.8 (15.6)	97.2 (18.0)	118 (22.3)	141.4 (33.4)	<0.001
TG, median (IQR), mg/dL	53.0 (42.0-69.0)	57.0 (46.0-76.0)	65.0 (49.0-83.0)	75.5 (58.0-99.0)	65.0 (58.0-92.0)	<0.001
HDL-C, mean (SD), mg/dL	62.9 (17.5)	61.9 (16.8)	62.4 (19.1)	56.5 (21.1)	62.0 (26.2)	0.012
Adulthood						
Age, mean (SD), y	45.7 (7.0)	46.2 (6.8)	45.9 (6.4)	43.6 (6.8)	44.5 (5.8)	0.002
BMI, mean (SD), kg/m^2	29.7 (7.3)	31.1 (7.7)	31.8 (7.4)	31.6 (7.7)	34.6 (7.9)	0.002
SBP, mean (SD), mm Hg	122 (17)	123 (17)	122 (15)	119 (16)	125 (13)	0.31
DBP, mean (SD), mm Hg	79 (11)	79 (11)	79 (10)	79 (12)	82 (10)	0.59
Glucose, mean (SD), mg/dL	99.8 (31.2)	104.8 (41.0)	106.3 (41.1)	102.4 (37.3)	125.7 (66.2)	0.03
TC, mean (SD), mg/dL	164.8 (28.0)	193.7 (33.1)	231.9 (40.8)	203.6 (35.4)	263.4 (64.6)	<0.001
non-HDL-C, mean (SD), mg/dL	108.8 (26.7)	141.9 (33.0)	183.9 (40.4)	155.6 (35.4)	211.3 (62.0)	<0.001
LDL-C, mean (SD), mg/dL	86.9 (22.0)	118.9 (27.2)	157.0 (31.0)	129.5 (27.5)	195.5 (75.7)	<0.001
TG, median (IQR), mg/dL	88.0 (65.0-131.0)	108.0 (76.0-152.0)	138.0 (92.0-208.0)	121.5 (84.0-181.0)	124.0 (88.0-194.0)	<0.001
HDL-C, mean (SD), mg/dL	54.5 (17)	50.7 (15.6)	48.3 (13.1)	47.7 (14.5)	49.7 (13.5)	<0.001
Lipid-lowing Medication, n (%)	13 (4.3)	64 (11.6)	33 (16.6)	33 (24.6)	7 (36.8)	<0.001
Hypertension, n (%)	112 (37.5)	212 (38.5)	83 (41.7)	54 (40.3)	13 (68.4)	0.10
Diabetes, n (%)	39 (13.0)	89 (16.2)	36 (18.1)	18 (13.4)	4 (21.1)	0.48
Smoking, n (%)	80 (26.8)	171 (31.1)	62 (31.2)	31 (23.1)	7 (36.8)	0.28
Alcohol drinking, n (%)	111 (37.1)	180 (32.7)	67 (33.7)	36 (26.9)	5 (26.3)	0.29
Carotid IMT, mean (SD), mm	0.844 (0.204)	0.929 (0.301)	0.969 (0.323)	0.906 (0.319)	1.044 (0.320)	<0.001

Abbreviations: BMI, body mass index; SBP, systolic blood pressure; DBP, diastolic blood pressure; TC, total cholesterol; non-HDL-C, Non-high-density lipoprotein-cholesterol; LDL-C, low-density lipoprotein cholesterol; HDL-C, high-density lipoprotein cholesterol; TG, triglycerides; IMT, intima-media thickness.

SI conversion factor: To convert glucose to mmol/L, multiply by 0.0555; to convert TC, non-HDL-C, LDL-C, and HDL-C to mmol/L, multiply by 0.0259; to convert TG to mmol/L, multiply by 0.0113.

© 2022 Yan Y et al. *JAMA Network Open.*

One-way ANOVA and chi-square tests were performed for continuous variables and categorical variables, respectively.

© 2022 Yan Y et al. *JAMA Network Open.*

eTable 4. Characteristics by TG Trajectory Group

Characteristics	Low-Stable (n=237)	Low-Slow Increase (n=505)	Low-Rapid Increase (n=191)	Moderate-Stable (n=202)	Moderate-Rapid Increase (n=66)	P
White, n (%)	112 (47.3)	333 (65.9)	144 (75.4)	165 (81.7)	55 (83.3)	<0.001
Male, n (%)	79 (33.3)	194 (38.4)	128 (67.0)	68 (33.7)	41 (62.1)	<0.001
Childhood						
Age, mean (SD), y	12.2 (4.5)	12.2 (4.4)	11.9 (4)	11.9 (4.7)	12.7 (5.2)	0.76
BMI, mean (SD), kg/m^2	18.3 (3.8)	18.9 (4.1)	18.7 (3.4)	20.3 (5.2)	20.6 (5.0)	<0.001
SBP, mean (SD), mm Hg	102 (12)	103 (11)	103 (11)	103 (11)	105 (10)	0.16
DBP, mean (SD), mm Hg	63 (10)	63 (10)	63 (10)	64 (10)	66 (9)	0.47
Glucose, mean (SD), mg/dL	85.8 (9.2)	86.7 (8.4)	87.5 (9.2)	87.1 (8.4)	91.7 (18.7)	<0.001
TC, mean (SD), mg/dL	149.1 (24.7)	153.5 (25)	153.8 (25.8)	168.6 (29.5)	162.3 (32.0)	<0.001
non-HDL-C, mean (SD), g/dL	82.7 (23.5)	89.8 (22.7)	90.9 (21.2)	115.0 (28.2)	112.5 (36.1)	<0.001
LDL-C, mean (SD), mg/dL	77.7 (22.0)	83.6 (20.9)	84.4 (19.8)	102.6 (25.8)	99.0 (30.2)	<0.001
TG, median (IQR), mg/dL	47.0 (38.0-58.0)	57.0 (46.0-71.0)	57.0 (47.0-74.0)	89.5 (68.0-114.0)	95.0 (77.0-111.0)	<0.001
HDL-C, mean (SD), mg/dL	66.5 (16.3)	63.7 (16.0)	62.9 (19.2)	53.6 (19.6)	49.8 (19.3)	<0.001
Adulthood						
Age, mean (SD), y	46.1 (6.4)	45.8 (7.0)	45.1 (7.0)	45.5 (6.5)	46.0 (7.0)	0.62
BMI, mean (SD), kg/m^2	29.3 (7.0)	30.6 (7.7)	31.9 (6.5)	32.2 (8.5)	33.6 (7.4)	<0.001
SBP, mean (SD), mm Hg	120 (17)	122 (17)	125 (15)	120 (16)	127 (16)	<0.001
DBP, mean (SD), mm Hg	77 (11)	79 (11)	81 (10)	79 (11)	83 (11)	<0.001
Glucose, mean (SD), mg/dL	96.6 (26.8)	100.4 (32.0)	113.3 (52.8)	102.5 (33.2)	134.3 (67.6)	<0.001
TC, mean (SD), mg/dL	181.2 (34)	192.1 (38.0)	200.5 (44.5)	201.9 (39.9)	226.4 (52.6)	<0.001
non-HDL-C, mean (SD), g/dL	121.5 (32.3)	139.8 (37.8)	156.4 (44.2)	150.8 (40.1)	185.7 (51.1)	<0.001
LDL-C, mean (SD), mg/dL	105.8 (30.3)	119 (34.1)	125.5 (40.4)	128.8 (42.6)	129.3 (42.0)	<0.001
TG, median (IQR), mg/dL	66.0 (53.0-81.0)	98.0 (77.0-126.0)	187.0 (136.0-235.0)	141.5 (105.0-176.5)	275.5 (207.0-350.0)	<0.001
HDL-C, mean (SD), mg/dL	58.9 (16.3)	51.4 (14.6)	44.8 (13.2)	49.8 (15.6)	39.1 (10.5)	<0.001
Lipid-lowing Medication, n (%)	20 (8.4)	50 (9.9)	29 (15.2)	33 (16.3)	18 (27.3)	<0.001
Hypertension, n (%)	76 (32.1)	201 (39.8)	83 (43.5)	79 (39.1)	35 (53.0)	0.02
Diabetes, n (%)	22 (9.3)	62 (12.3)	44 (23.0)	34 (16.8)	24 (36.4)	<0.001
Smoking, n (%)	47 (19.8)	151 (29.9)	57 (29.8)	73 (36.1)	23 (34.8)	0.003
Alcohol drinking, n (%)	69 (29.1)	175 (34.7)	68 (35.6)	69 (34.2)	18 (27.3)	0.42
Carotid IMT, mean (SD), mm	0.851 (0.204)	0.907 (0.271)	0.956 (0.285)	0.936 (0.268)	0.994 (0.375)	<0.001

Abbreviations: BMI, body mass index; SBP, systolic blood pressure; DBP, diastolic blood pressure; TC, total cholesterol;

© 2022 Yan Y et al. *JAMA Network Open.*

non-HDL-C, Non-high-density lipoprotein-cholesterol; LDL-C, low-density lipoprotein cholesterol; HDL-C, high-density lipoprotein cholesterol; TG, triglycerides; IMT, intima-media thickness.

SI conversion factor: To convert glucose to mmol/L, multiply by 0.0555; to convert TC, non-HDL-C, LDL-C, and HDL-C to mmol/L, multiply by 0.0259; to convert TG to mmol/L, multiply by 0.0113.

One-way ANOVA and chi-square tests were performed for continuous variables and categorical variables, respectively.

© 2022 Yan Y et al. *JAMA Network Open.*

eTable 5. Characteristics by HDL-C Trajectory Group

Characteristics	Low-Stable (n=226)	Moderate-Slow Decrease (n=606)	Moderate-Stable (n=137)	High-Rapid Decrease (n=182)	High-Stable (n=50)	P
White, n (%)	186 (82.3)	426 (70.3)	78 (56.9)	99 (54.4)	20 (40.0)	<0.001
Male, n (%)	114 (50.4)	266 (43.9)	30 (21.9)	85 (46.7)	15 (30.0)	<0.001
Childhood						
Age, mean (SD), y	11.6 (4.4)	11.9 (4.5)	12.2 (4.7)	13.3 (4.2)	12.5 (4.6)	0.002
BMI, mean (SD), kg/m^2	20.1 (5.1)	18.9 (4.3)	18.6 (3.5)	19.0 (3.6)	18.2 (3.1)	0.001
SBP, mean (SD), mm Hg	104 (11)	102 (11)	101 (10)	104 (11)	106 (14)	0.04
DBP, mean (SD), mm Hg	64 (10)	62 (10)	64 (10)	65 (10)	68 (12)	<0.001
Glucose, mean (SD), mg/dL	86.2 (8.9)	87.5 (10.3)	85.3 (8.6)	87.9 (9.1)	85.4 (8.0)	0.03
TC, mean (SD), mg/dL	150.5 (28)	153.3 (26.3)	158.8 (26.5)	163.2 (25.5)	172.8 (26.5)	<0.001
non-HDL-C, mean (SD), g/dL	107.7 (30.9)	92.6 (25.2)	92.4 (25.1)	85.2 (22.7)	87.5 (26.0)	<0.001
LDL-C, mean (SD), mg/dL	96.1 (27.2)	85.2 (22.3)	86.6 (24.0)	80.6 (21.5)	82.4 (24.4)	<0.001
TG, median (IQR), mg/dL	77.0 (57.0-106.0)	59.0 (46.0-76.0)	58.0 (46.0-74.0)	51.0 (42.0-63.0)	55.0 (44.0-68.0)	<0.001
HDL-C, mean (SD), mg/dL	42.7 (13.8)	60.8 (13.5)	66.4 (13.7)	78 (15.6)	85.3 (14.4)	<0.001
Adulthood						
Age, mean (SD), y	45.0 (7.0)	45.3 (6.8)	46.7 (5.9)	46.8 (7.0)	46.8 (6.3)	0.009
BMI, mean (SD), kg/m^2	32.8 (7.7)	31 (7.8)	28.2 (6.8)	31.3 (6.9)	29.2 (5.8)	<0.001
SBP, mean (SD), mm Hg	123 (15)	122 (17)	120 (16)	122 (15)	120 (16)	0.39
DBP, mean (SD), mm Hg	80 (11)	79 (11)	78 (12)	78 (10)	80 (10)	0.31
Glucose, mean (SD), mg/dL	108.8 (41.7)	104.7 (38)	93.7 (39.9)	105.0 (40.8)	95.9 (20.2)	0.004
TC, mean (SD), mg/dL	187.6 (42.9)	194.0 (40.0)	205.8 (41.5)	194.5 (40.4)	202.6 (31.3)	0.006
non-HDL-C, mean (SD), g/dL	147.4 (42.8)	146.2 (40.8)	132.7 (43.5)	139.0 (42.0)	125.6 (33.7)	0.001
LDL-C, mean (SD), mg/dL	118.5 (36.6)	121.6 (37.5)	113.0 (36.0)	120.5 (38.6)	112.2 (33.0)	0.09
TG, median (IQR), mg/dL	141.0 (103.0-209.0)	107.0 (77.0-156.0)	81.5 (63.5-125.0)	102.5 (74.0-143.0)	82.0 (58.0-112.5)	<0.001
HDL-C, mean (SD), mg/dL	39.6 (9.7)	47.2 (10.9)	71.7 (15.5)	54.6 (11.3)	76.3 (16.6)	<0.001
Lipid-lowing medication, n (%)	36 (15.9)	73 (12.0)	9 (6.6)	26 (14.3)	6 (12.0)	0.11
Hypertension, n (%)	94 (41.6)	238 (39.3)	51 (37.2)	74 (40.7)	17 (34.0)	0.83
Diabetes, n (%)	52 (23.0)	94 (15.5)	8 (5.8)	28 (15.4)	4 (8.0)	<0.001
Smoking, n (%)	71 (31.4)	186 (30.7)	36 (26.3)	49 (26.9)	9 (18.0)	0.26
Alcohol drinking, n (%)	63 (27.9)	209 (34.5)	60 (43.8)	50 (27.5)	17 (34.0)	0.01
Carotid IMT, mean (SD), mm	0.975 (0.366)	0.917 (0.296)	0.879 (0.241)	0.892 (0.201)	0.851 (0.166)	0.03

© 2022 Yan Y et al. *JAMA Network Open.*

Abbreviations: BMI, body mass index; SBP, systolic blood pressure; DBP, diastolic blood pressure; TC, total cholesterol; non-HDL-C, Non-high-density lipoprotein-cholesterol; LDL-C, low-density lipoprotein cholesterol; HDL-C, high-density lipoprotein cholesterol; TG, triglycerides; IMT, intima-media thickness.

SI conversion factor: To convert glucose to mmol/L, multiply by 0.0555; to convert TC, non-HDL-C, LDL-C, and HDL-C to mmol/L, multiply by 0.0259; to convert TG to mmol/L, multiply by 0.0113.

One-way ANOVA and chi-square tests were performed for continuous variables and categorical variables, respectively.

© 2022 Yan Y et al. *JAMA Network Open.*

eTable 6. Covariate-Adjusted Carotid IMT and P Values for Comparisons Between Lipid Trajectory Groups

Trajectory group	Mean IMT (SE)[a]	Trajectory groups			
		1	2	3	4
TC					
1. Low-Stable	0.887 (0.017)	Ref			
2. Low-Slow Increase	0.932 (0.012)	0.02	Ref		
3. Low-Rapid Increase	0.952 (0.021)	0.01	0.38	Ref	
4. Moderate-Stable	0.926 (0.021)	0.13	0.81	0.37	Ref
5. High-Stable	1.039 (0.045)	0.001	0.02	0.07	0.02
non-HDL-C					
1. Low-Stable	0.876 (0.016)	Ref			
2. Low-Slow Increase	0.924 (0.012)	0.009	Ref		
3. Low-Rapid Increase	0.972 (0.023)	<0.001	0.05	Ref	
4. Moderate-Stable	0.988 (0.021)	<0.001	0.006	0.61	Ref
5. High-Stable	1.107 (0.066)	<0.001	0.006	0.05	0.08
LDL-C					
1. Low-Stable	0.867 (0.016)	Ref			
2. Low-Slow Increase	0.935 (0.012)	<0.001	Ref		
3. Low-Rapid Increase	0.970 (0.019)	<0.001	0.10	Ref	
4. Moderate-Stable	0.959 (0.023)	<0.001	0.34	0.69	Ref
5. High-Stable	1.034 (0.060)	0.007	0.10	0.31	0.24
TG					
1. Low-Stable	0.885 (0.018)	Ref			
2. Low-Slow Increase	0.927 (0.013)	0.05	Ref		
3. Low-Rapid Increase	0.946 (0.020)	0.03	0.40	Ref	
4. Moderate-Stable	0.956 (0.020)	0.007	0.18	0.71	Ref
5. Moderate-Rapid increase	0.942 (0.034)	0.15	0.67	0.91	0.70
HDL-C					
1. High-Stable	0.886 (0.038)	Ref			
2. High-Rapid Decrease	0.889 (0.020)	0.17	Ref		
3. Moderate-Stable	0.918 (0.023)	0.46	0.55	Ref	
4. Moderate-Slow Decrease	0.933 (0.012)	0.07	0.05	0.35	Ref
5. Low-Stable	0.962 (0.019)	0.08	0.93	0.14	0.23

SE, standard error; TC, total cholesterol; non-HDL-C, non-high-density lipoprotein-cholesterol; LDL-C, low-density lipoprotein cholesterol; HDL-C, high-density lipoprotein cholesterol; TG, triglycerides; IMT, intima-media thickness

a, adjusted for age, race, sex, and body mass index, lipid-lowering medication, hypertension, diabetes, smoking, alcohol drinking, and lipid levels in the last adult survey.

© 2022 Yan Y et al. *JAMA Network Open.*

eTable 7. Covariate-Adjusted Means of Adult Carotid IMT by Lipid Trajectory Group Adjusted for Clinical Risk Factors and Lipid Levels at Follow-up

Trajectory group	Mean IMT (SE)[a]	P
TC		
Low-Stable	0.916 (0.025)	Ref
Low-Slow Increase	0.974 (0.016)	0.04
Low-Rapid Increase	0.959 (0.030)	0.31
Moderate-Stable	0.962 (0.027)	0.20
High-Stable	1.086 (0.060)	0.01
non-HDL-C		
Low-Stable	0.901 (0.023)	Ref
Low-Slow Increase	0.964 (0.016)	0.02
Low-Rapid Increase	0.988 (0.033)	0.04
Moderate-Stable	1.042 (0.029)	<0.001
High-Stable	1.129 (0.089)	0.02
LDL-C		
Low-Stable	0.872 (0.018)	Ref
Low-Slow Increase	0.932 (0.012)	0.004
Low-Rapid Increase	0.966 (0.022)	0.002
Moderate-Stable	0.960 (0.024)	0.003
High-Stable	1.017 (0.063)	0.03
TG		
Low-Stable	0.882 (0.021)	Ref
Low-Slow Increase	0.926 (0.013)	0.05
Low-Rapid Increase	0.949 (0.022)	0.04
Moderate-Stable	0.957 (0.020)	0.01
Moderate-Rapid Increase	0.947 (0.037)[a]	0.16
HDL-C		
Low-Stable	0.955 (0.020)	0.26
Moderate-Slow Decrease	0.930 (0.012)	0.49
Moderate-Stable	0.930 (0.026)	0.50
High-Rapid Decrease	0.892 (0.020)	0.86
High-Stable	0.900 (0.042)	Ref

SE, standard error; TC, total cholesterol; non-HDL-C, non-high-density lipoprotein-cholesterol; LDL-C, low-density lipoprotein cholesterol; HDL-C, high-density lipoprotein cholesterol; TG, triglycerides; IMT, intima-media thickness

a, adjusted for age, race, sex, and body mass index, lipid-lowering medication, hypertension, diabetes, smoking, alcohol drinking, and lipid levels in the last adult survey.

© 2022 Yan Y et al. *JAMA Network Open.*

eTable 8. Covariate-Adjusted Means of Adult Carotid IMT by Lipid Trajectory Group After Excluding Participants With Lipid-Lowing Medications

Trajectory group	Model 1			Model 2	
	Mean (SE)	P		Mean (SE)	P
TC					
Low-Stable	0.873 (0.016)	Ref		0.871 (0.019)	Ref
Low-Slow Increase	0.913 (0.012)	0.03		0.913 (0.012)	0.04
Low-Rapid Increase	0.943 (0.021)	0.007		0.944 (0.022)	0.02
Moderate-Stable	0.912 (0.022)	0.14		0.914 (0.025)	0.19
High-Stable	1.030 (0.050)	0.002		1.035 (0.055)	0.007
non-HDL-C					
Low-Stable	0.865 (0.015)	Ref		0.860 (0.017)	Ref
Low-Slow Increase	0.910 (0.012)	0.01		0.910 (0.012)	0.01
Low-Rapid Increase	0.957 (0.024)	0.001		0.960 (0.025)	0.001
Moderate-Stable	0.968 (0.022)	<0.001		0.977 (0.026)	<0.001
High-Stable	1.083 (0.077)	0.005		1.101 (0.082)	0.005
LDL-C					
Low-Stable	0.860 (0.015)	Ref		0.863 (0.017)	Ref
Low-Slow Increase	0.921 (0.012)	<0.001		0.921 (0.012)	0.004
Low-Rapid Increase	0.945 (0.020)	<0.001		0.943 (0.020)	0.004
Moderate-Stable	0.937 (0.025)	0.006		0.932 (0.029)	0.06
High-Stable	0.981 (0.070)	0.09		0.971 (0.074)	0.17
TG					
Low-Stable	0.872 (0.018)	Ref		0.876 (0.019)	Ref
Low-Slow Increase	0.915 (0.013)	0.04		0.916 (0.013)	0.06
Low-Rapid Increase	0.913 (0.020)	0.13		0.913 (0.020)	0.18
Moderate-Stable	0.933 (0.020)	0.02		0.928 (0.022)	0.08
Moderate-Rapid Increase	0.942 (0.037)	0.09		0.936 (0.038)	0.18
HDL-C					
Low-Stable	0.927 (0.019)	0.22		0.927 (0.022)	0.28
Moderate-Slow Decrease	0.920 (0.012)	0.26		0.920 (0.012)	0.28
Moderate-Stable	0.899 (0.023)	0.58		0.899 (0.023)	0.59
High-Rapid Decrease	0.875 (0.020)	0.98		0.874 (0.022)	0.98
High-Stable	0.876 (0.037)	Ref		0.875 (0.040)	Ref

SE, standard error; TC, total cholesterol; non-HDL-C, non-high-density lipoprotein-cholesterol; LDL-C, low-density lipoprotein cholesterol; HDL-C, high-density lipoprotein cholesterol; TG, triglycerides; IMT, intima-media thickness

Model 1, adjusted for age, race, sex, and body mass index, lipid-lowering medication, hypertension, diabetes, smoking, alcohol drinking, and lipid levels in the last adult survey.

Model 2, Model 1 plus adjusted for lipid levels at baseline

© 2022 Yan Y et al. *JAMA Network Open.*

eFigure 1. Flow Chart of the Study Cohort Selection

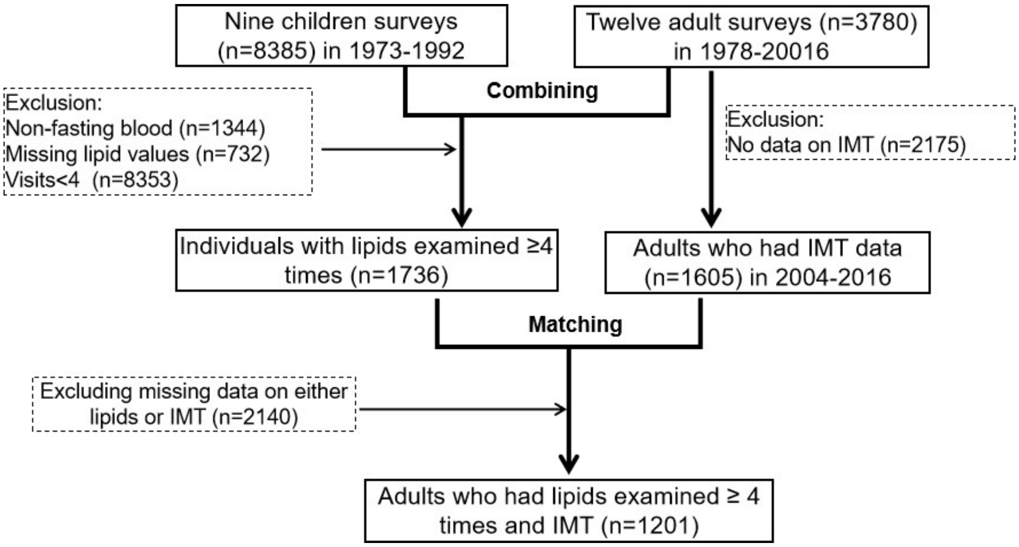

© 2022 Yan Y et al. *JAMA Network Open.*

eFigure 2. Growth Curves of Lipids by Race and Sex

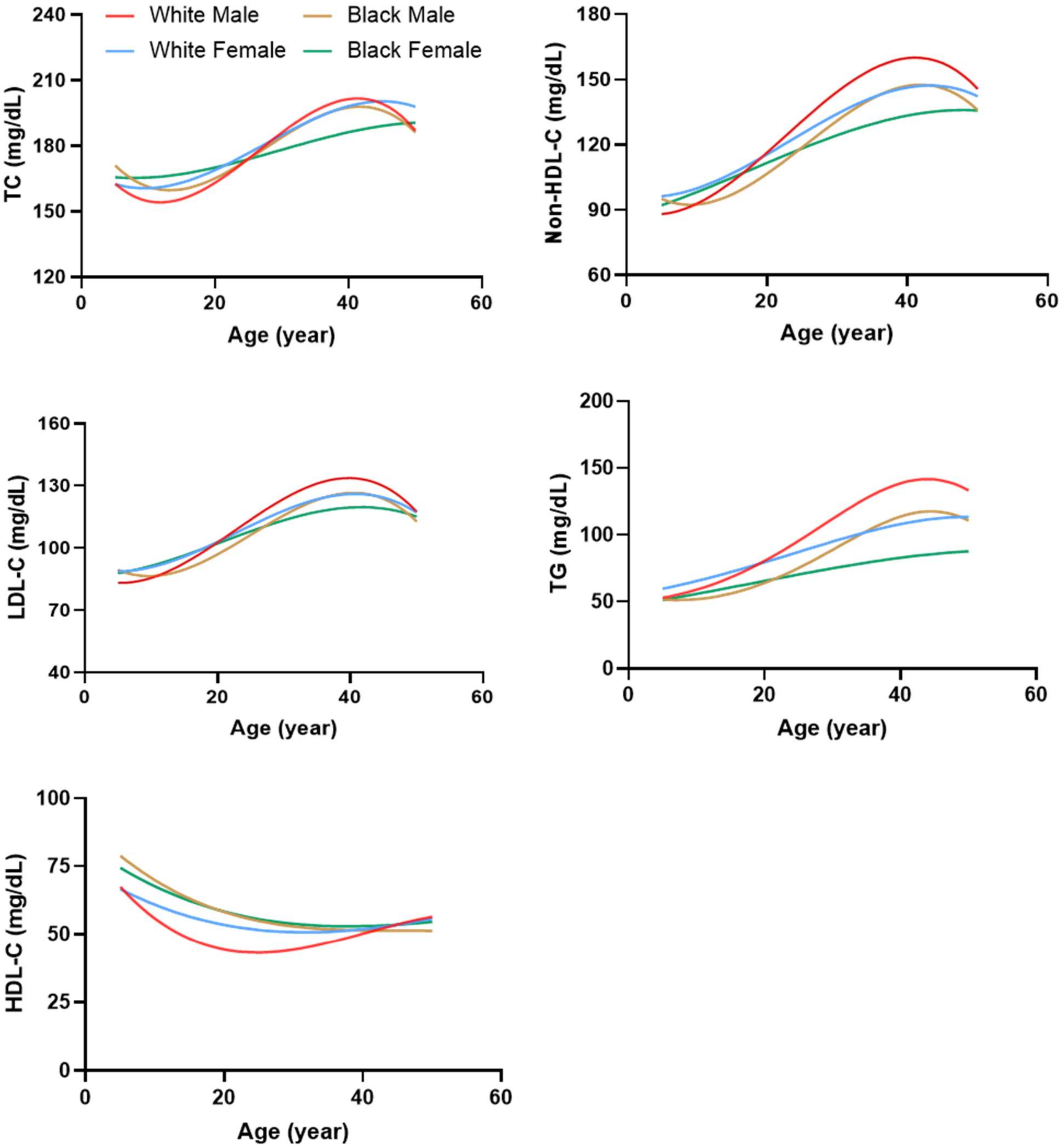

TC, total cholesterol; non-HDL-C, non-high-density lipoprotein-cholesterol; LDL-C, low-density lipoprotein cholesterol; TG, triglycerides; HDL-C, high-density lipoprotein cholesterol

© 2022 Yan Y et al. *JAMA Network Open.*
